# Burden of socio-legal concerns among vulnerable patients seeking cancer care services at an urban safety-net hospital: a cross-sectional survey

**DOI:** 10.1186/s12913-016-1443-1

**Published:** 2016-06-14

**Authors:** Naomi Yu Ko, Tracy A. Battaglia, Rebecca Gupta-Lawrence, Jessica Schiller, Christine Gunn, Kate Festa, Kerrie Nelson, JoHanna Flacks, Samantha J. Morton, Jennifer E. Rosen

**Affiliations:** Section of Hematology Oncology, Women’s Health Unit, Boston Medical Center, 801 Massachusetts Ave, First Floor, Boston, MA 02118 USA; Women’s Health Unit, Boston Medical Center, 801 Massachusetts Ave, First Floor, Boston, MA 02118 USA; Department of Biostatistics, Boston University, 801 Massachusetts Avenue, 3rd Floor, Boston, MA 02118 USA; Medical-Legal Partnership I Boston, c/o Nutter McClennen & Fish LLP 155 Seaport Blvd, Boston, MA 02210 USA; Department of Surgery, MedStar Washington Hospital Center, 110 Irving Street NW Suite G247C, Washington DC, 20010 USA

**Keywords:** Cancer, Safety net, Legal partnerships, Disparities

## Abstract

**Background:**

Social and economic conditions that affect one’s ability to satisfy life’s most basic needs such as lack of affordable housing, restricted access to education and employment, or inadequate income are increasingly well-documented barriers to optimal health. The burden of these challenges among vulnerable patients accessing cancer care services is unknown.

**Methods:**

We conducted a cross-sectional survey of patients presenting for ambulatory cancer care services (screening and treatment) at an urban safety-net hospital to assess socio-legal concerns (social problems related to meeting life’s basic needs supported by public policy or programming and potentially remedied through legal advocacy/action).

**Results:**

Among 104 respondents, 80 (77 %) reported concerns with one or more socio-legal needs in the past month, with a mean of 5.75 concerns per participant. The most common socio-legal concerns related to income supports, housing, and employment/education.

**Conclusion:**

Our findings support the need for innovations in cancer care delivery to address socio-legal concerns of a vulnerable patient population.

**Electronic supplementary material:**

The online version of this article (doi:10.1186/s12913-016-1443-1) contains supplementary material, which is available to authorized users.

## Background

It is well-established that social environments of the poor and underserved negatively influence health [1, 2]. In particular, poor and underserved populations across the United States continue to bear a disproportionate burden of cancer, including higher incidence [3], advanced stage at diagnosis [4, 5], and higher mortality [3, 5, 6]. While the interplay of race/ethnicity, poverty and other socioeconomic indicators is complex, research clearly has documented the adverse impact that the social determinants of health can have on the ability to access and engage in timely and quality cancer care services [3, 7–9]. Despite state government (i.e., Massachusetts) attempts to offer uniform access to care, we still see persistent disparities which suggest that challenges are multifactorial and can originate outside the health care delivery setting. Indeed, a host of community-specific social, cultural, behavioral, and systems barriers to cancer care are commonly found among those living in poverty [5, 10]. These barriers are repeatedly found to affect health outcomes negatively for these populations [3, 11].

Federal and state laws in the United States have established a range of programs, services, and protections intended to address a host of socioeconomic vulnerabilities such as inadequate nutrition, unsafe and unaffordable housing, and insufficient income. Using this “social safety net”, qualified people may be eligible for a variety of health-promoting benefits and services [12]. Efforts by the health care delivery system to ensure adequate individual and population health are undermined when patients do not receive the protections or benefits that these laws afford them. Despite the existence of safety-net programs such as Supplemental Nutritional Assistance Program (SNAP) [13], Section 8 housing subsidies [14–17], and Supplemental Security Income (SSI) [18] (among many others), inconsistent program implementation coupled with complex bureaucratic requirements can result in unlawful denial of benefits and services, leading to preventable poor health outcomes [19, 20]. Landlords, school districts, and employers often fail to comply with a range of legal obligations they have to ensure, for example, non-discrimination in housing, education, and the workplace. The federal government has made recent attempts to address these social determinants of health in order to reduce disparities [21–26]. Despite enthusiasm and focus on rethinking how to engage health care professionals in the social determinants of health [27], health care providers are not traditionally trained to identify or address health-affecting socio-legal concerns nor do they typically have appropriate resources available to them within their care team. This resource gap has become more pronounced in recent years as historically scarce social work resources are increasingly dedicated to mental health counseling rather than more traditional social work advocacy (i.e., food stamps, social support resources, arranging for additional resources) [28].

Medical-legal partnership (MLP) strategies were developed in the context of such needs. The MLP model first was implemented in 1993 to serve the pediatric population at Boston Medical Center. Lawyers were integrated into the health care team to address legal problems that affected the health of vulnerable inner-city children and their families [19]. As the model evolved, MLP efforts were targeted to five key domains of socio-legal concerns: Income Supports; Housing and Utilities; Education and Employment; Legal Status (immigration); and Personal and Family Stability and Safety [19]. Early successes in Boston triggered national expansion; [29] currently, 276 hospitals and health centers in 36 states and the District of Columbia incorporate medical-legal partnerships into their care for low-income patients [30]. We worked with Medical-Legal Partnership | Boston, the founding site of the national MLP Network, to conduct this study because of the program’s long history serving patient-families treated at Boston Medical Center.

To date, literature documenting the burden of socio-legal concerns exists mainly for pediatric populations [19, 31–34]. The data relating to cancer patients is limited in that it (a) resides mainly in legal literature in the form of case studies [35–37] or as program reports [31, 38, 39], (b) is qualitative in nature [35], and/or (c) does not target the most vulnerable subpopulations of cancer patients [19, 36, 38–40]. A systematic effort to identify and understand socio-legal concerns within the cancer care patient population is critical to inform care delivery for those most at risk for poor cancer outcomes. The purpose of this study is to describe the prevalence of a range of socio-legal concerns in a diverse low-income population seeking cancer care services at an urban safety-net hospital.

## Methods

The Boston University Institutional Review Board approved this cross-sectional study. We used a research-assistant administered questionnaire to measure the prevalence and nature of socio-legal concerns among patients seeking ambulatory cancer care services at an inner-city safety-net academic medical center from September 2010 to June 2011.

### Socio-legal concerns

While the term “social determinants of health” is widespread in academic medical and public health literature, we sought to evaluate whether patients would report socioeconomic indicators that are potentially remediable under existing law and public policy, as this could be suggestive of a range of potential interventions depending on the findings. We defined the term socio-legal concerns as concerns about adverse socioeconomic conditions that have potential legal remedies residing in laws, regulations, or public policies [40, 41]. For example, a patient lacking enough food to eat may have been unlawfully denied government nutrition benefits—such as the Supplemental Nutrition Assistance Program—to which s/he was legally entitled. Here, the need for adequate nutrition required for good health becomes a socio-legal concern because this patient’s access to certain nutritional benefits is prescribed by law and can be enforced through legal advocacy. Our findings are limited to the prevalence of socio-legal concerns in a particular population; this study did not test a particular intervention. We use the phrase socio-legal concerns (as opposed to, for instance, legal concerns) for two reasons:

1. Socioeconomic conditions receptive to resolution exist on a continuum of acuity—what may present as a legal situation (e.g., a patient confronting an eviction notice that could potentially trigger homelessness) likely earlier existed in a less acute posture (e.g., that same patient being behind on his rent for several months).
2. Some socioeconomic hardships simultaneously trigger legal risks (e.g., eviction) and remedies enshrined in the law that can only be exercised through downstream engagement with court processes, or through upstream engagement with administrative agency bureaucracy(ies) (e.g., successful application for public benefits that boost income available for rent).

Across the broad spectrum of socioeconomic adversities with potential legal remedies, some material hardships lend themselves especially well to preventive (upstream) approaches that can, for example, keep lights and heat on before the risk escalates to service disconnection with attendant health implications. The authors’ preferred term, socio-legal concerns, reflects their recognition that social determinants of health can have complex trajectories, meaning that a range of responsive interventions could be implicated at different stages of that risk/problem/barrier life cycle.

### Population studied

We included patients seeking care in three ambulatory cancer practice sites in one inner-city safety-net hospital, in order to capture the concerns of patients across the entire cancer care spectrum, from screening through diagnosis and treatment. Clinical sites included a breast specialty practice in which patients are referred for screening and diagnostic services, a medical oncology clinic, and a same-day surgery clinic for cancer patients in active treatment. Eligible participants were aged 18 or older and able to speak and read English. There were no restrictions on sex, ethnicity, primary language, insurance status, or type or stage of cancer. However, the sites where we recruited had a majority of female patients seeing breast cancer screening and treatment services.

### Survey instrument

A survey was developed to identify socio-legal concerns that may act as barriers to cancer care [41]. The following three sources informed survey design: 1) a literature review on those social determinants of health observed among low-income cancer patients; [9, 12, 35, 37, 42, 43] 2) existing questions from instruments utilized by MLP | Boston [44] in 2009–10 to identify patients in need of legal help; and 3) a previously validated questionnaire used to identify families in need of legal help [10]. Questions were formulated to fit within five core socio-legal concern categories, reflected in the acronym I-HELP^SM^. Each category reflects basic needs and factors that affect health and are amenable to direct intervention, including: Income Supports, Housing and Utilities, Education/Employment, Legal Status, and Personal and Family Stability and Safety [45]. A total of 24 questions were included across the five I-HELP categories, and participants were systematically asked “In the last month, have you been concerned about the following…” A “yes” response to one or more of these 24 questions was considered a positive response for the presences of a socio-legal concern. Survey instrument can be found as an Additional file 1.

Additional survey questions included: Health status using the single SF-12 question rating for health (excellent, good, fair, poor); [46] the reason for the participant’s medical visit along the cancer care continuum (screening, diagnosis, treatment related); and the short four-question version of Cohen, Kamarck & Mermelstein’s Perceived Stress Scale [38]. A 10-item Demographics section completed the survey. The survey was originally piloted with 15 individual patients and modified based on feedback and research group consensus.

### Data collection

We enrolled a sample of consecutive eligible patients arriving for ambulatory visits. After permission from one of three providers (surgical oncologist, primary care physician and medical oncologist), research assistants approached patients in clinic waiting rooms or exam rooms before/during their scheduled appointments and invited them to participate. Those interested in participation gave verbal consent, and the survey was administered in a private location. Each question and the subsequent response options were read aloud. Responses were recorded directly by pen and paper onto the questionnaire by the research assistant (responses were subsequently entered into an Excel spreadsheet and transferred into SAS). Once the survey was complete, if any legal concerns were identified through the survey interaction, literature was provided with contact information of an appropriate person or agency.

### Data analysis

Descriptive statistics were performed on patient demographics, health status, and socio-legal concerns using percentages for categorical factors, and mean (standard deviation), or median (interquartile range) for non-normally distributed continuous factors. Comparisons of the groups of participants reporting any socio-legal concerns and participants reporting no socio-legal concerns were conducted using chi-squared tests and t-tests for significance. We examined type of socio-legal concern by frequency and number of unique patients who responded positive to each specific concern. SAS v 9.0 was used to perform analysis [47].

## Results

A total of 197 patients were screened for eligibility after arrival to a cancer care-related ambulatory clinic visit and 73 were found to be ineligible (non-English speaking status *n* = 46, or non-cancer-related reason for their visit *n* = 16). Among the 124 eligible patients invited to participate, a total of 104 participants completed the I-HELP survey (84 % response rate). Table 1 displays participant socio-demographic characteristics by participant socio-legal concern status. The mean age of all study participants was 49 years (range 18–85), 65 % were from racial or ethnic minority groups, 67 % had an education of high school or less, 43 % had an annual household income of less than $20,000, and 63 % were enrolled in public health insurance. Overall, 80 participants (77 %) reported concerns about one or more socio-legal need in the last month. Unadjusted chi-squared analyses revealed that those who reported one or more socio-legal concerns were more likely to be: Black, single, and low-income (*p* < .05). Participants with socio-legal concerns have higher stress on average as measured by the Perceived Stress Scale [38] (mean 9.40) compared to those with no legal concerns (mean 7.23, *p* < .004). See Table 1.

**Table 1:** Patient Characteristics by Socio-Legal Concern (*N* = 104)

	Total	No Socio-Legal Concerns	≥1 Socio-Legal Concerns	P-value
	104	24 (23 %)	80 (77 %)	
Age (yr), mean, range	49 (18–85)	52 (21–85)	48 (18–84)	0.33
Gender, n (%)				0.67
Female	73 (70)	67 %	71 %	
Male	31 (30)	33 %	29 %	
Race, n (%)				0.09
Black/African-American	50 (48)	29 %	54 %	
White	36 (35)	50 %	30 %	
Other	18 (17)	21 %	16 %	
Non-English Primary Language^a^, n (%)	40 (38)	38 %	39 %	0.91
Marital Status, n (%)				0.04
Single	43 (41)	21 %	48 %	
Married/Partnered	37 (36)	54 %	30 %	
Divorced/Separated/Widowed	24 (23)	25 %	23 %	
Education, n (%)				0.12
High School or Less	70 (67)	54 %	71 %	
College or Grad School	34 (33)	46 %	29 %	
HH Income^a^, n (%)				0.03
< $20,000	45 (43)	29 %	48 %	
$20,000–$50,000	20 (19)	8 %	23 %	
> $50,000	22 (21)	46 %	14 %	
Has Primary Care Physician^a^, n (%)	97 (93)	88 %	95 %	0.35
Health Insurance^a^, n (%)				0.16
Public^b^	65 (63)	33 %	54 %	
Private	35 (34)	42 %	29 %	
Type of Visit, n (%)				0.94
Screening/Diagnostic eval	44 (42)	42 %	43 %	
Treatment	60 (58)	58 %	58 %	
Perceived Stress (mean, SD)^c^	8.92 (3.23)	7.23 (2.37)	9.40 (3.29)	0.004
Overall Health (mean, SD)^d^	2.24 (0.91)	1.96 (0.69)	2.35 (0.95)	0.09

^a^Percentage points may not add up to 100 % due to missing data

^b^Includes Health SafetyNet, MAHealth, Commonwealth, Celtic Care

^c^Score from the Perceived Stress Scale [1], a 4 question survey where scores around 13 are considered average, a higher score means greater stress

^d^Score from the RAND Medical Outcomes Study, 12-item Short Form Survey [2]. Scores 50 and above represent average health status

Table 2 provides a detailed analysis of the individual responses to survey questions corresponding with the I-HELP categories. Overall, the top reported socio-legal concern was “having enough money to pay basic expenses” (53 %) followed by “concern with finances” (38 %). Among specific concerns with income supports and public benefits, nearly a third of participants report concerns with getting government benefits or disability benefits, paying for their prescriptions, or obtaining adequate health insurance coverage. Among housing concerns, cost of housing concerns dominated, followed closely by worries about housing conditions and safety in the home. Education concerns regarding participant’s children (29 %) were as commonly reported as concerns regarding the participants’ own employment (26 %).

**Table 2:** Number and frequency of socio-legal concerns amongst 104 participants reporting ≥ 1 social-legal concern within the last month (*N* = 460)

IHELP	Specific Concern	N (%)
	In the past month, have you been concerned about…	
I	…having enough money to pay for your basic expenses?	55 (53 %)
I	…finances (credit, bankruptcy, taxes, auto/housing insurance & medical expenses)?	39 (38 %)
I	…being able to afford prescription drugs and other medical expenses?	32 (31 %)
I	…having health insurance for you or your family members?	28 (28 %)
I	…getting government benefits and services for your family?	27 (28 %)
I	…applying for or receiving disability benefits?	24 (24 %)
I	…having enough food (including any special needs) to eat?	13 (13 %)
I	…finding affordable and reliable childcare?	6 (15 %)
H	…the cost of your housing?	33 (33 %)
H	…being able to pay utility bills (electric & heating)?	33 (33 %)
H	…the safety or condition of your housing?	21 (20 %)
H	…being able to find housing in a safe neighborhood?	16 (30 %)
H	…being evicted or unable to pay your mortgage?	16 (17 %)
H	…being discriminated against in your housing search?	4 (7 %)
E	…employment?	23 (26 %)
E	…your children’s education?	14 (29 %)
E	…your children’s safety when at school?	10 (22 %)
L	…your family’s immigration status?	7 (12 %)
P	…family safety?	13 (13 %)
P	…family violence?	7 (7 %)
O	…making decisions for your future if you become ill or injured?	26 (25 %)
O	…care for elderly or disabled relatives?	12 (14 %)
O	…problems with police, jail or criminal justice system?	1 (1 %)

Only 28 of those with reported socio-legal concerns (35 %) responded that “yes” they had discussed the socio-legal concern with their health care providers. Among those respondents, only 16 (20 %) reported that they had support from legal services, patient advocacy, or social work to address them (not in table).

Table 3 displays the frequency of reported socio-legal concerns across the I-HELP categories overall and by unique participant. Among the 80 participants who reported concerns with one or more socio-legal concerns, a combined total of 460 socio-legal concerns were reported corresponding with a mean of 5.75 concerns per participant (range 1–17). Nearly all survey participants with socio-legal concerns (90 %) reported at least one concern related to income supports and public benefits, while two-thirds (66 %) reported concerns with housing, and 41 % reported concerns with their employment or their own (or their child’s) education. Fewer participants reported concern with legal status (immigration) (9 %) or personal/family stability and safety (19 %). Thirty-three participants (41 %) also reported other socio-legal concerns that did not fit into these categories, including such things as advanced directives (*n* = 26) and caring for elderly/disabled relatives (*n* = 12).

**Table 3:** Frequency of Reported Socio-Legal Concerns by I-HELP Categories (*N* = 460 unique concerns reported by *N* = 104 participants)

	Total number of socio-legal concerns reported	Number of unique participants with >1 or more socio-legal concern	Mean number of socio-legal concerns per participant	Median number of socio-legal concerns per participant
	N (%)	N (%)	(range)	(SD)
I - Income Supports	224 (47 %)	72 (90 %)	2.80 (0–7)	2 (2.02)
H - Housing	123 (27 %)	53 (66 %)	1.54 (0–6)	1 (1.58)
E - Education/Employment	47 (10 %)	33 (41 %)	0.59 (0–3)	0 (0.84)
L - Legal Status	7 (1 %)	7 (9 %)	0.09 (0–1)	0 (0.28)
P - Personal/Family Stability & Safety	20 (4 %)	15 (19 %)	0.25 (0–2)	0 (0.97)
O - Other	39 (8 %)	33 (41 %)	0.49 (0–2)	0 (0.49)
Total	460	80	6.11 (1–18)	5 (4.33)

## Discussion

This observational study is the first to provide a systematic assessment of the socio-legal concerns among a vulnerable population seeking care across the cancer care spectrum. Not only did we find a high percentage of patients reporting socio-legal concerns (77 %), but multiple socio-legal concerns were present, with a mean of 5.75 concerns per patient. The most common concerns reported are income-or housing-related which highlights the potential impact that material hardship may play among cancer patients living in poverty, even in the face of expanded access to health coverage. Also striking is that our findings suggest that socio-legal concerns are associated with a higher level of stress and these concerns are not being discussed with care providers. This represents a missed opportunity in terms of referral for advice and assistance, and the potential overall health care quality improvement such ancillary services may engender.

Assessing socio-legal concerns is an innovative way to quantify upstream social determinants of health [42], described as personal resources such as education and income and the social environments in which people live, work, study, and engage in recreational activities [12]. Woolf and Braveman describe how these contextual conditions influence people’s exposure to environmental risks and their personal health behaviors, vulnerability to illness, access to care, and ability to manage conditions at home [12, 42]. As the health equity movement begins to shed light on the significance of social determinants of health, there is growing evidence of basic needs going unmet where eligible people are not connected to legally-prescribed benefits and services [36]. While this can be traced to many factors, one potential modifiable variable is the prevalence of unlawful denials of benefits and services or failure to enforce existing laws. Understanding how or which of these barriers might culminate to create barriers to obtaining needed health care services and achieving health outcomes. Further research in the impact of the social determinants of health will be critical to understanding and potentially improving population health.

Our study findings are consistent with other findings ascertained using different methodologies. The most recent assessment of unmet legal needs by the American Bar Association concludes that low-income Americans typically have 2–3 civil legal needs in areas related to health, including housing, disability supports, family stability and safety, access to health coverage and access to disability accommodations in work and school [30–37, 42]. Retkin et al. performed a cross-sectional study of 51 cancer patients, and 61 % reported two or more legal issues, including financial concerns, disability issues, health insurance, workplace issues or advanced care planning. Only 20 % reported that they had their needs addressed by a health care provider [31]. In a 2006 survey of a largely White population of 2,307 cancer survivors, 75 % of respondents said they had experienced one or more practical concerns, where that term was defined as: school issues, employment issues, debt, or insurance [39]. Another study utilizing focus group data from 50 cancer patients identified 30 legal issues and documented a significant impact on quality of life [35]. In 2010, the American Cancer Society Cancer Action Network supported the conduct of a nationally representative sample of 1,011 adults who reported that they or their household member had a history of cancer. Half of those surveyed reported difficulty paying for health care costs, one-third had difficulty paying for basic life necessities, and 23 % delayed care because of costs [38]. Our study is the first to assess these types of concerns systematically among an ambulatory inner city safety-net population.

The high prevalence of income and housing concerns in this population [1] is especially troubling given the current economic environment, where the average income of Americans has been declining since 1999, while food insecurity, unaffordable housing costs and homelessness are increasing [48–53]. This growing income and social mobility gap threatens to widen cancer disparities. If a patient is having trouble accessing a health-promoting public benefit to which s/he is entitled, this needs to be detected. Our data provide a new framework to capture the burden of socio-legal concerns for patients seeking cancer-related services across the entire cancer care continuum in a racially and ethnically diverse, low-income, safety-net population. Our findings suggest that these concerns are not routinely disclosed to health care providers and thus represent a missed opportunity for addressing a range of high-stakes needs. Using a systematic method to capture and potentially act on socio-legal concerns in a vulnerable patient population is an innovative method of improving health care delivery.

The call for reorientation of health care systems to the early identification of social determinants [27] is opening a new frontier in addressing the non-biological factors that profoundly influence health; [1, 27] proactively to identify and then meet the needs of individual cancer patients throughout their cancer journey. Our study findings suggest that innovations in care delivery to address these socio-legal concerns are warranted. Interventions underway that merit rigorous evaluation include patient navigation, oncology social work, and medical-legal partnership based in cancer care.

We note that in the last decade or so, there has been substantial growth in integration of legal advocates and attorneys as newer members of the cancer care team [36]. Many such programs are affiliated with the National Cancer Legal Services Network (convened by LegalHealth, a division of the New York Legal Assistance Group) and the national MLP network (convened by the National Center for Medical-Legal Partnership, based at George Washington University Milken Institute School of Public Health) [30, 31].

In recent years, more evidence is building for medical-legal partnerships across the health care spectrum. For example, Ryan, et al. [48] surveyed participants in a family medicine clinic for stress levels before and after receiving medical-legal services. This recent pilot study demonstrated that participants had overall 30 % less stress and a 41 % increase in sense of well-being after receiving care from a medical-legal partnership. These improvements in levels of stress and well-being are particularly relevant to our study given our finding of higher levels of stress in our participants who had socio-legal concerns. Overall, emerging evidence suggests that the presence of an MLP intervention in any health care setting holds promise as one approach to address concerns identified by our vulnerable patients seeking cancer services. Future evidence that identifies health related consequences due to socio-legal concerns can help to establish the impact of our findings.

### Limitations

We recognize several limitations of our findings. 1) The inclusion of only English-speaking subjects limits the generalizability of our findings, yet doing so likely underestimates the true burden of socio-legal concerns, since non-English speaking patients are at even higher risk for living in poverty [54, 55]. Future work that includes multi-lingual speakers will be critical. 2) Our findings represent only patients seeking care at a single institution in Massachusetts, where health care reform leaves few without insurance and where housing costs in the Northeast may create a disproportionately high level of housing concerns than might not be reflected in other U.S. communities. One might reasonably expect that the type of socio-legal concerns present in a given community reflect the public policy and socioeconomic climate in that given community, so that these findings may not be a good guide to specific needs in another community. 3) Using a dichotomous response scale for the survey limits our ability to understand the continuum of socio-economic conditions. 4) By directly administering surveys, we were able to ensure completion of data in a low literacy population, but we also acknowledge this is at the expense of a social desirability bias. 5) There may also be a response bias in our population due to a possible over or under estimating of the burden of socio-legal concerns as we do not have knowledge of these concerns in individuals who did not enroll into the study. 6) This study is limited to a population of only females and a single cancer type (breast) without representation from individuals who are in active radiation treatment.

## Conclusion

Social determinants of health often are a root cause of illness and are key to understanding health disparities. Some of these determinants can be addressed through existing law and public policy. The data presented here provide a new lens through which to evaluate, approach, and remedy cancer health disparities in our country. Among a vulnerable inner city population seeking cancer care services, we demonstrate a high prevalence of socio-legal concerns. Given these findings and the consensus that social determinants of health are difficult and costly to address, we must continue the investigation by evaluating the possible impact of interventions that address socio-legal concerns, including but not limited to medical-legal partnership [42].

## Abbreviations

I-HELP, income supports, housing and utilities, education/employment, legal status, and personal; MLP, medical-legal partnership; SNAP, supplemental nutritional assistance program; SSI, supplemental security income

## Additional file

Additional file 1: Survey Instrument. Copy of administered survey instrument to identify socio-legal concerns that may act as barriers to cancer care. Questions were formulated to fit within five core socio-legal concern categories: Income Supports, Housing and Utilities, Education/Employment, Legal Status, and Personal and Family Stability and Safety. A total of 25 questions were included across the five I-HELP categories. (PDF 180 kb)
