# Supplementary material for: Burden of socio-legal concerns among vulnerable patients seeking cancer care services at an urban safety-net hospital: a cross-sectional survey
Source: BMC Health Serv Res. 2016 Jun 14;16:196. doi: 10.1186/s12913-016-1443-1 (PMC4906581; doi:10.1186/s12913-016-1443-1)
Supplement: Additional file 1: — Survey Instrument. Copy of administered survey instrument to identify socio-legal concerns that may act as barriers to cancer care. Questions were formulated to fit within five core socio-legal concern categories: Income Supports, Housing and Utilities, Education/Employment, Legal Status, and Personal and Family Stability and Safety. A total of 25 questions were included across the five I-HELP categories. (PDF 180 kb) [file 12913_2016_1443_MOESM1_ESM.pdf]

BMC Patient Medical-Legal Need Survey©

Section 1: Health Status

1. How do you rate your health?

- □ Excellent
- □ Good
- □ Fair
- □ Poor

2. Why are you here today?

- □ Screening
- □ Follow-up on Abnormal Screening
- □ Cancer Treatment
- □ Follow-up on Cancer Treatment
- □ Other (please specify)____

3. What is your diagnosis? ____

For Research Use Only:

□ AIDS Malignancy	□ Breast	□ Head and Neck	□ Multiple Myeloma
□ Amyloidosis	□ Gastrointestinal	□ Leukemia	□ Sarcoma
□ Gynecological	□ Genitourinary	□ Lung	□ Unknown
□ Brain	□ Lymphoma	□ Melanoma	□ Other

4. When were you diagnosed?

- □ <3 months
- □ 3-6 months ago
- □ 7-12 months ago
- □ > 1 year ago

Section 2: DEMOGRAPHIC INFORMATION

5. How old are you? ______

6. Sex: □ Female □ Male □ Transgender

7. Which of the following best describes you? (Check all that apply)

- □ Hispanic/Latino
- □ American Indian/Alaska Native
- □ White
- □ Unknown
- □ Asian
- □ Native Hawaiian or Other Pacific Islander
- □ Black or African American
- □ Other (please specify)_____________________

8. In what country were you born?_____________________________

9. What other languages do you speak? ____

10. Education: (Check highest education completed)

- □ Grade School (Primary School)
- □ High School (Secondary School)
- □ College (University)
- □ Graduate School (Professional School)

Copyrighted material 2016: not for use without authors' permission

11. Marital Status: (Check one)

□ Single
□ Married
□ Separated
□ Partnered
□ Divorced
□ Widowed

12. What is your annual household income? (Check one)

□ < $10,000
□ $10,000-19,999
□ $20,000-49,999
□ $50,000-$99,999
□ > $100,000

13. Do you have a primary care physician? If yes, where?

□ **Yes, at Boston Medical Center.**
□ **Yes, at a Community Health Clinic.**
□ **Yes, Other**
□ **No, I do not have a primary care physician.**

14. How many adults (age 21+), including you, live in your home right now? ________

15. How many children (ages 0-20) live in your home right now? ___________

Section 3: MEDICAL AND LEGAL NEEDS

16. For the following questions please check off only one answer. Your responses will be kept confidential.

**In the last month, have you been concerned about the following….**	YES	NO	N/A
a)…the safety or condition of your housing?			
b)…the cost of your housing?			
c)…being evicted or unable to pay your mortgage?			
d)…being discriminated against in your housing search?			
e)…being able to find housing in a safe neighborhood?			
f)…getting government benefits and services for your family?			
g)...having enough money to pay for your basic expenses?			
h)…finances (credit, bankruptcy, taxes, auto/housing insurance & medical expenses?			
i)…being able to pay utility bills (electric & heating)?			
j)…having enough food (including any special needs) to eat?			
k)…employment?			
l)…your family's immigration status?			
m)…your children's education?			
n)…your children's safety when at school?			

Copyrighted material 2016: not for use without authors' permission

In the last month, have you been concerned about the following….	YES	NO	N/A
o)…finding affordable and reliable childcare?			
p)…having health insurance for your or your family members?			
q)…family safety?			
r)…family violence?			
s)…dealing with a new illness or injury?			
t)…being able to afford prescription drugs and other medical expenses?			
u)…applying for or receiving disability benefits?			
v)…making decisions for your future if you become ill or injured?			
w)…care for elderly or disabled relatives?			
x)…problems with police, jail or criminal justice system?			

17. **Have any of these legal needs impacted your health?** If yes, please describe.

(1)☐ **Yes**

(2)☐ **No**

18. **Have you spoken with a health care provider about any of these legal needs?** If yes, please describe.

(1)☐ **Yes**

(2)☐ **No**

19. **Have you made use of any of the following?** (Check all that apply)

(1)□ Social worker
(2)□ Legal aid services at the hospital
(3)□ Legal aid services outside of the hospital
(4)□ Patient advocate
(5)□ Other
(6)□ None of the above

20. **Does anyone in your family receive government benefits including food stamps, WIC, welfare (TAFDC), emergency assistance (EAEDC), SSI/SSDI, military benefits, or unemployment?** If yes, please describe.

(1)☐ **Yes**

(2)☐ **No**

21. **Which of the following best describes your housing situation?** (Check one)

(1)□ Homeless
(2)□ Adult Emergency Shelter
(3)□ Renting an apartment at market-rent cost
(4)□ Renting room in a home or boarding house
(5)□ Family Shelter
(6)□ Public Housing (projects or Section 8)
(7)□ Own Home
(8)□ Other

Copyrighted material 2016: not for use without authors' permission

22. How do you pay for your health care? (Check all that apply)

(1)□ Private insurance (e.g. Blue Cross, Tufts, Harvard Pilgrim, Commonwealth Choice)
(2)□ Health safety net (formerly "free care")/MassHealth Limited
(3)□ MassHealth (NOT MassHealth Limited)
(4)□ Commonwealth Care or Celtic Care
(5)□ Medicare
(6)□ I have no health insurance.
(7)□ Other (please specify)_______________
(8)□ Unknown

23. Approximately how much money do you or your family spend on average on medical coverage (including monthly premiums and out-of-pocket costs) per year?

(1)□ less than 50 dollars
(2)□ between 50 and 99 dollars
(3)□ between 100 and 499 dollars
(4)□ between 500 and 999 dollars
(5)□ over 1000 dollars
(6)□ other
(7)□ unknown

24. Have you been denied medical insurance coverage? If yes, please describe.

(1)☐ **Yes**

(2)☐ **No**

25. Have you had any problems with communication around medical treatment and appointments? Please explain: (Mail / Phone?)

(1)☐ **Yes**

(2)☐ **No**

26. For the following questions please check off only one answer. *Your responses will be kept confidential.*

**In the last month, how often have you….**	Never	Rarely	Sometimes	Most of the time	All of the time	Not Applicable
a)…felt that you were unable to control the important things in your life?						
b)...felt confident about your ability to handle your personal problems?						
c)…felt that things were going your way?						
d)…felt difficulties were piling up so high that you could not overcome them?						

Thank you very much for your cooperation!

Copyrighted material 2016: not for use without authors' permission
